# Repetitive Transcranial Magnetic Stimulation Ameliorates Cognitive Impairment by Enhancing Neurogenesis and Suppressing Apoptosis in the Hippocampus in Rats with Ischemic Stroke

**DOI:** 10.3389/fphys.2017.00559

**Published:** 2017-08-02

**Authors:** Feng Guo, Jicheng Lou, Xiaohua Han, Yuguo Deng, Xiaolin Huang

**Affiliations:** ^1^Department of Rehabilitation Medicine, Tongji Hospital, Tongji Medical College, Huazhong University of Science and Technology Wuhan, China; ^2^Department of Obstetrics and Gynecology, The Central Hospital of Wuhan Wuhan, China

**Keywords:** rTMS, focal cerebral ischemia, cognitive impairment, hippocampal neurogenesis, BDNF signaling pathway

## Abstract

Cognitive impairment is a serious mental deficit caused by stroke that can severely affect the quality of a survivor's life. Repetitive transcranial magnetic stimulation (rTMS) is a well-known rehabilitation modality that has been reported to exert neuroprotective effects after cerebral ischemic injury. In the present study, we evaluated the therapeutic efficacy of rTMS against post-stroke cognitive impairment (PSCI) and investigated the mechanisms underlying its effects in a middle cerebral artery occlusion (MCAO) rat model. The results showed that rTMS ameliorated cognitive deficits and tended to reduce the sizes of cerebral lesions. In addition, rTMS significantly improved cognitive function via a mechanism involving increased neurogenesis and decreased apoptosis in the ipsilateral hippocampus. Moreover, brain-derived neurotrophic factor (BDNF) and its receptor, tropomyosin-related kinase B (TrkB), were clearly upregulated in ischemic hippocampi after treatment with rTMS. Additionally, further studies demonstrated that rTMS markedly enhanced the expression of the apoptosis-related B cell lymphoma/leukemia gene 2 (Bcl-2) and decreased the expression of the Bcl-2-associated protein X (Bax) and the number of TUNEL-positive cells in the ischemic hippocampus. Both protein levels and mRNA levels were investigated. Our findings suggest that after ischemic stroke, treatment with rTMS promoted the functional recovery of cognitive impairments by inhibiting apoptosis and enhancing neurogenesis in the hippocampus and that this mechanism might be mediated by the BDNF signaling pathway.

## Introduction

Stroke is now the third leading cause of death worldwide and the leading cause of death and disability in China (Yang et al., 2013; Herrington et al., 2015). The consequences of stroke include significant deficits in both motor and cognitive function, which continue to represent a medical challenge and a large socioeconomic burden. Several studies have reported that up to 30% of stroke survivors suffer cognitive impairments within 3 months (Al-Qazzaz et al., 2014). In addition, stroke is the second most common cause of cognitive impairment behind neurological disease (Qu et al., 2015). Despite its high prevalence, no unequivocally efficacious drug is currently available to treat PSCI.

The hippocampus is a functional region of the brain that is involved in human cognition. The hippocampus is especially important for spatial learning and memory. Hippocampal lesions caused by a stroke or secondary damage caused by a focal ischemic stroke contribute to the pathogenesis of PSCI (Grysiewicz and Gorelick, 2012; Tang et al., 2012). An increasing amount of evidence indicates that neurogenesis in the hippocampus is important for cognitive functions (Kitamura and Inokuchi, 2014; Suarez-Pereira and Carrion, 2015). In the subgranular zone (SGZ), neural stem cells (NSCs) give rise to neuroblasts, which mature into functional dentate granule neurons that become integral prats of the pre-existing hippocampal circuitry (Mathews et al., 2010). In addition, cognitive impairment is strongly associated with neuronal apoptosis, which is tightly regulated by a variety of signal transduction cascades (Feng et al., 2013; Kim et al., 2016). These insights suggest that approaches aimed at activating endogenous NSCs may stimulate the generation of new neurons and inhibit neuronal apoptosis. When applied following an ischemic event in the adult hippocampus, these types of therapies could have clinical potential in PSCI.

rTMS is a noninvasive brain-stimulating technique with potential neuroprotective activity that has recently received an increasing amount of interest (Nierat et al., 2015). Some studies have found that exposure to rTMS stimulates the proliferation of adult NSCs (Zhang et al., 2014) and inhibits apoptosis in peri-ischemic tissues (Gao et al., 2010). However, these findings may not be applicable in cases in which the hippocampus has been damaged by stroke, and few studies have focused on PSCI. We therefore performed an intensive research study to investigate the effect of rTMS on PSCI and to explore the mechanisms underlying these effects. We view this technique as critical for developing better therapies for cerebral ischemia patients.

Several signaling molecules have been demonstrated to be essential for neurogenesis (Faigle and Song, 2013), which has been associated with improved cognitive functions. The signaling pathway involving BDNF and its receptor, TrkB, has been shown to play important roles in both the developing and adult central nervous system, including participation in process such as synaptic plasticity, learning, and neurogenesis (Rossi et al., 2006; Camerino et al., 2016). Manipulations that activate BDNF signaling in neurogenic regions may be beneficial if they enhance neurogenesis and cognitive recovery after focal cerebral ischemia. Previous studies have shown that rTMS modulates BDNF signaling in cultured hippocampal neurons (Ma et al., 2013) obtained from mice as well as human lymphocytes and human cortical tissues (Wang et al., 2011). Thus, it is possible that rTMS could be used to promote adult neurogenesis by affecting BDNF signaling to exert a neurorehabilitory effect in PSCI. In addition, to further explore the protective role of rTMS in ischemic stroke, we examined the expression of the apoptosis-related proteins Bcl-2 and Bax in the ischemic hippocampus.

The present study was designed to investigate the effect of 10 Hz rTMS on cognitive dysfunction following focal cerebral ischemia. Our results confirm that the rTMS-induced neuroprotective effect is associated with changes in apoptosis and the expression of BDNF signaling pathway components, which cooperatively regulate hippocampal neurogenesis. These findings reveal a potential mechanism by which rTMS may improve cognitive impairment, indicating that rTMS is a promising candidate for the development of clinical strategies to treat ischemic stroke.

## Materials and methods

### Animals

Adult male *Sprague-Dawley* rats (230–260 g; Jingda Bioengineering Co., Ltd., Hunan, China) were used in this study. The rats were housed under a 12-h light/dark cycle and provided *ad libitum* access to food and water. All experimental procedures were approved by the ethics committee of the Tongji Medical College (Permit Number: 298), and all animal treatments were performed strictly in accordance with the Care and Use of Laboratory Animals of the National Institutes of Health Guide. The utmost effort was made to minimize the number of animals used and their suffering.

### Experimental design and groups

The rats were randomly divided into 7- and 14-day groups, each of which was further divided into Sham, MCAO, and rTMS groups. Apart from the Sham group, the remaining groups underwent MCAO surgery, and only the rTMS group received rTMS treatment.

### MCAO procedures

The rats were anesthetized using 10% chloral hydrate (400 mg/kg, i.p.). The right middle cerebral artery was occluded for 90 min and subsequently reperfused (Longa et al., 1989). Surgery was induced using the intraluminal suture occlusion method as previously described (Lin et al., 2013). During the surgical procedures, rectal temperature was maintained at 37 ± 0.5°C using a heat lamp. In the Sham group, only the external carotid artery was ligated.

### Magnetic resonance imaging (MRI)

All rats were examined using a Discovery MR750 3.0 T magnetic resonance imaging (MRI) scanner (GE, USA) equipped with a radio frequency (RF) coil for animals. MRI was performed at 24 h, 7 and 14 days after surgery. Each rat was deeply anesthetized and then placed in a prone position with its head in the middle of the coil. Diffusion-weighted images (DWI) were obtained using a two-dimensional spin-echo echo-planar imaging sequence (SE-EPI) using the following parameters: 2,000/minimum; field of view, 50 × 5 mm; thickness, 2 mm and image acquisition matrix, 96 × 64. The infarct volume was expressed as a percentage (%) of the whole brain volume. Apparent diffusion coefficient (ADC) maps were calculated from the DWI data. The values of the ADCs were then measured for the ischemic slice and the mirrored contralateral region. Lesion volumes (LV) were evaluated from ADC images as the ratio of the ischemic region to the corresponding region in the contralateral hemisphere (mean ± *SD*; Bar-Shir et al., 2010; Huang et al., 2013).

### rTMS treatment

rTMS was delivered using a customized magnetic stimulator (YRD-CCI, Wuhan, China). In conscious rats, a round prototype coil (6 cm in diameter with 3.5T peak magnetic welds) was positioned perpendicular to the cortex ~5 mm to the right of bregma (Linden et al., 1999). The treatment protocol consisted of stimulation for 3 s followed by rest for 50 s and was repeated 10 times (300 pulses per day) at a rate of 10 Hz. The stimulation intensity was set at 120% of the average resting motor threshold (RMT). All procedures were based on previous studies involving animal experiments (Guo et al., 2014).

### Administration of bromodeoxyuridine

Bromodeoxyuridine (BrdU) (50 mg/kg in saline, Sigma-Aldrich, USA) was intraperitoneally injected once daily for 7 consecutive days starting 24 h after surgery in all groups (*n* = 5 for each group; Chern et al., 2014).

### Morris water maze task

Learning and memory performance was assessed using the Morris water maze test beginning on the 12th day after surgery. The principles and technical details of the task have been described previously (Han et al., 2013). Briefly, a circular water tank (150 cm in diameter and 50 cm deep) was filled with 23 ± 2°C water to a depth of 21 cm. A circular platform 10 cm in diameter and 20 cm in height was placed in the center of the target quadrant (quadrant III). Several visual cues were placed on the walls of the test room. The rats were subjected to two sessions of four place navigation trials per day at intervals of at least 4 h for 2 consecutive days (day 12 through day 13, and the mice were trained 4 times). The starting points were changed for every trial. On day 14, the platform was removed for the spatial probe trial. The latency to find the submerged platform, the frequency of swimming across the platform and the swimming paths taken were automatically recorded using a computer-based image analyzer (MWM tracking system MT-200, ChengDu Technology & Market Co., Ltd., Chengdu, Sichuan Province, China). All data were recorded by the same person, who was blind to the grouping of the rats.

### Tissue preparation

To avoid any potential impact on the final results, we excluded rats that exhibited abnormal behavioral effects, such as seizures, during treatment. All rats were deeply anesthetized using 10% chloral hydrate (400 mg/kg, i.p.) at different time points. A subset of the rats was transcardially perfused with 0.9% NaCl followed by 4% paraformaldehyde in 0.1 M phosphate-buffered saline (PBS). The brains of these rats were removed and post-fixed in the same fixative at 4°C overnight and then immersed consecutively in 20 and 30% sucrose at 4°C until they sank. The brains of rats injected with BrdU (*n* = 10 in each group) were cut into 30-μm-thick consecutive coronal sections (from 2.15 to 5.76 mm behind bregma) and prepared for immunofluorescence staining. The remaining post-fixed brains (*n* = 5 in each group) were embedded in paraffin and then cut into 10-μm-thick coronal sections (from 2.15 to 5.76 mm behind bregma). These sections were used for TUNEL staining. The rats in which the brains were not fixed were immediately sacrificed, and the hippocampus ipsilateral to the treatment was collected for western blot and RT-PCR analyses.

### Immunofluorescence staining and image processing

Frozen 30-μm-thick sections were incubated in blocking solution (10% normal donkey serum or 10% normal goat serum and 0.3% Triton X-100 in PBS, pH 7.5) for 2 h at room temperature (RT) and then incubated with primary antibodies in 5% serum and PBS for 24 h at 4°C. The sections were subsequently incubated for 3 h at RT with fluorophore-conjugated secondary antibodies. To prepare the sections for BrdU staining, they were incubated in 2 N HCl for 0.5 h at 37°C.

Rat monoclonal anti-BrdU (1:100; Abcam, UK) primary antibodies were used to mark proliferating cells, mouse monoclonal anti-Nestin (1:100; BD Pharmingen, USA) primary antibodies were used as a marker for NSCs, and mouse monoclonal anti-NeuN (1:100; Chemicon, USA) primary antibodies were used to label mature neurons. The following secondary antibodies were used: Alexa Fluor 594-labeled donkey anti-rat IgG and Alexa Fluor 488-labeled donkey anti-mouse IgG (1:200 for both; Invitrogen, USA).

The stained slides were dehydrated, cover-slipped in anti-quenching agent (p-phenylenediamine, PPD) and analyzed using a confocal laser-scanning microscope (Olympus, Tokyo, Japan). The number of positive cells was counted in a blinded manner in the ipsilateral SGZ using 20X and 40X objectives in OLYMPUS FV10-ASW Viewer.

### TUNEL staining

Frozen 10-μm-thick sections were prepared for TUNEL staining. *In situ* apoptosis was analyzed using a TUNEL assay kit (*In situ* Cell Death Detection Kit; Roche, USA) according to the manufacturer's instructions. Coded slides were initially examined for the presence TUNEL-positive cells in subfields of the hippocampus. The percentage of cells that were positive for TUNEL staining was then estimated (Kim et al., 2014). A quantitative analysis of the percentages of viable neurons was performed using Image-Pro Plus (Leica DMLB) software at 400x magnification. An average of three Tunel-stained sections was analyzed to yield a single parameter per rat. The examiner was blind to the group assignment of each animal.

### Western blot analysis

Anti-rabbit BDNF (1:1,000; Santa Cruz, Inc., CA, USA), TrkB (1:1,000; Santa Cruz, Inc., CA, USA), p-AKT (1:1,000; Santa Cruz, Inc., CA, USA), Bcl-2 (1:1,000, Cell Signaling Technology, Inc., MA, USA), and Bax (1:1,000, Cell Signaling Technology, Inc., MA, USA) antibodies were used. Images were acquired using an X-ray film processor. Normalization was performed using mouse monoclonal GAPDH antibodies (1:500; Santa Cruz, Inc., CA, USA). The bands were quantitated using Gel-Pro Analyzer 4.0 software (Media Cybernetics, USA).

### RT-PCR analysis

Real-time reverse-transcription polymerase chain reaction (RT-PCR) was used to analyze the expression levels of the BDNF and TrkB mRNAs in ipsilateral hippocampal tissues. RT-PCR was performed in duplicate for each RNA sample. Glyceraldehyde-3-phosphate dehydrogenase (GAPDH) was used as an internal control. The fold-changes in BDNF and TrkB expression were calculated relative to GAPDH expression using the comparative Ct method (2^−ΔΔCT^). mRNA levels were then expressed as fold-changes after normalization to GAPDH levels.

### Statistical analysis

All data are presented as the mean ± *SD* and were analyzed using SPSS 20.0 (IBM Corporation, Somers, NY, USA). Statistical comparisons of results were performed using one-way ANOVA. The Bonferroni correction was used to account for multiple tests. *P* < 0.05 was regarded as statistically significant, and all numerical analyses were performed using Graph Pad Prism.

## Results

### Effect of rTMS on lesion volume ratios after focal cerebral ischemia in rats

**Figure 1:**
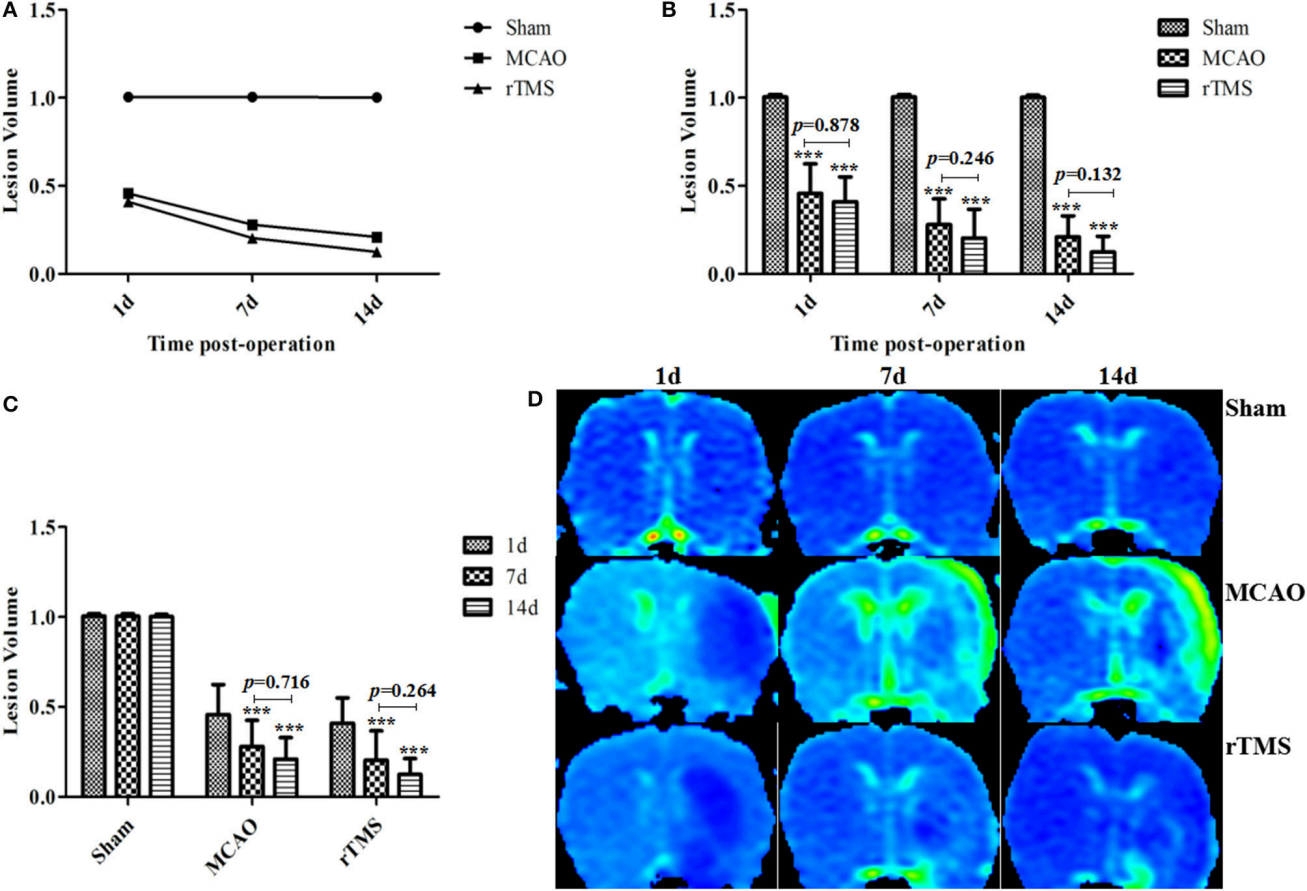
Effect of rTMS on lesion volume ratios in ischemic rats at 1, 7, and 14 d after surgery. **(A)** The percentage LV was calculated from ADC maps. **(B)** Comparisons among the three groups at different time points. **(C)** Comparisons among the three time points within each group. **(D)** Representative ADC maps for the results obtained at 1 d, 7 d, and 14 d post-occlusion in the three groups. ^***^P < 0.001 compared to the Sham group.

Lesion volumes (LV) were evaluated from ADC images as the ratio of the ischemic region to the corresponding region in the contralateral hemisphere of the rats. We calculated LV for all groups on the first day, 7th day, and 14th day post-occlusion (Figure 1A). In the Sham group, ADC signals were similar between the cerebral hemispheres (LV = 1.004 ± 0.012) during the experiment.

First, we compared the results in the three groups at different time points (Figure 1B). At 1, 7, and 14 d post-occlusion, the LV were lower in the MCAO and rTMS rats than in the Sham group (*P* < 0.001). An analysis of variance showed that LV was initially, at 1 d post-occlusion, very similar between the MCAO (0.456 ± 0.16) group and the rTMS (0.410 ± 0.134) group (*P* = 0.878). While the difference in LV values between the two non-Sham groups was also non-significant on both on day 7 (MCAO: 0.279 ± 0.14, rTMS: 0.203 ± 0.156, *P* = 0.246) and day 14 (MCAO: 0.209 ± 0.114, rTMS: 0.124 ± 0.085, *P* = 0.132) post-MCAO, the tissues appeared to show more improvement in the rTMS group than in the MCAO group.

We performed an additional analysis to determine whether there were differences among the groups at different time points (Figure 1C). The results showed that there was no significant difference in LV between the values obtained on day 7 and day 14 post-surgery in both the MCAO (*P* = 0.716) group and the rTMS (*P* = 0.264) group. This might be because pseudo-normalization generally occurs on the 7th day following this treatment (Yang et al., 2015). Representative ADC maps for the three groups on days 1, 7, and 14 post-occlusion are shown in Figure 1D.

### Effects of rTMS on cognitive function after focal cerebral ischemia

**Figure 2:**
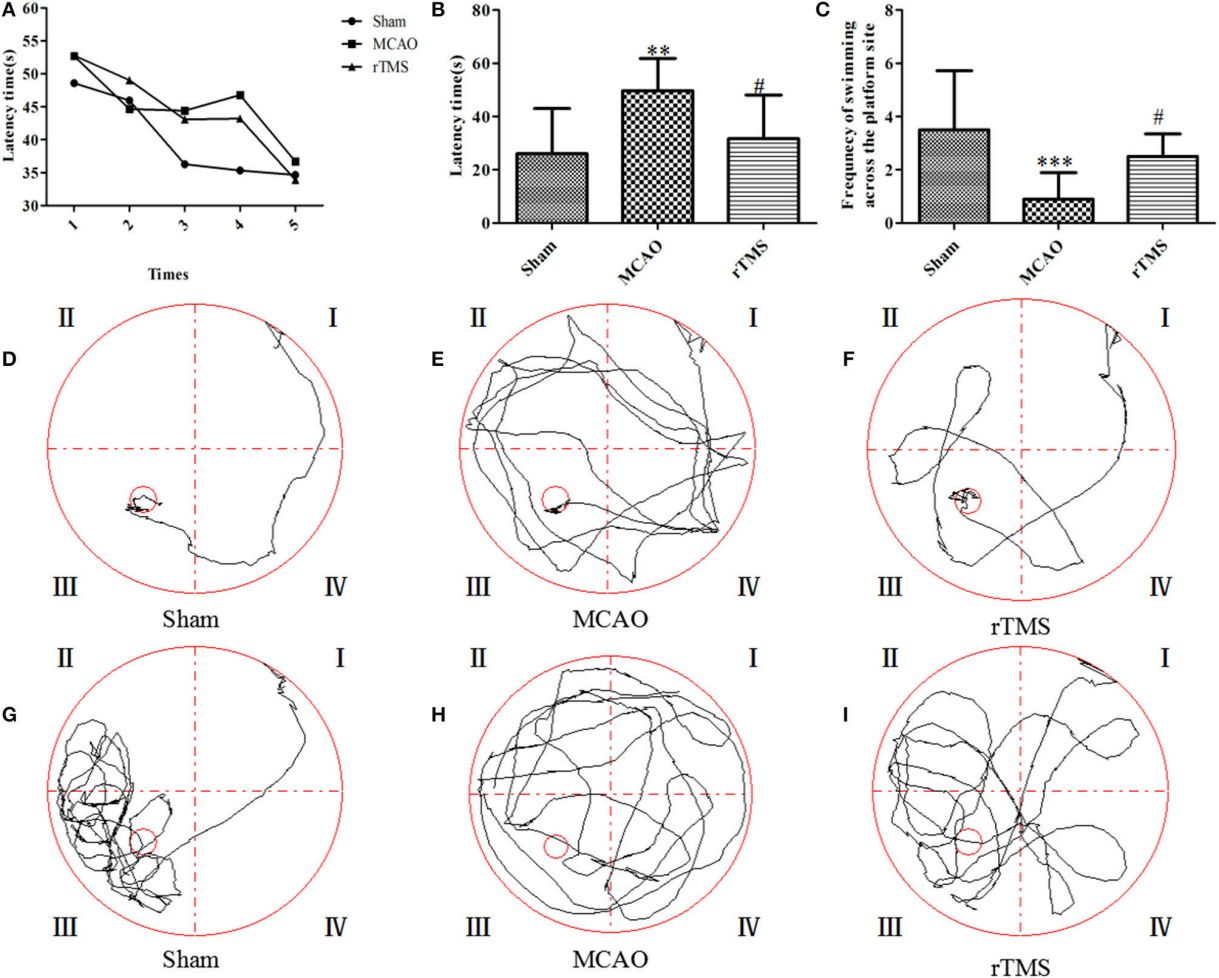
Effects of rTMS on cognitive impairment in the Morris water maze task. **(A,B)** Latencies to find the submerged platform from the 1st time to the 5th time. **(C)** The frequency of swimming across the platform site in the spatial probe trial. **(D–F)** Representative track plots of each group. **(G–I)** Representative track plots of the frequency of swimming across the platform in the spatial probe trials. The gray lines indicate the swimming traces. ^**^P = 0.006 and ^***^P = 0.002 for the MCAO group vs. the Sham group; ^#^*P* < 0.05 for the rTMS group vs. the MCAO group.

Cognitive performance was assessed by averaging the values for latency to reach the platform and counting the frequency of swimming across the platform in the spatial probe trial. An analysis of escape latencies showed that there were significant differences among the Sham, MCAO, and rTMS groups. Escape latency was longer (*P* = 0.042) in the MCAO group (49.7 ± 12.16 s) than in the Sham group (26 ± 17.04 s) and shorter (*P* = 0.006) in the rTMS group (31.7 ± 16.38 s) than in the MCAO group (Figures 2A,B). The typical swimming paths observed in each group are shown in Figures 2D–F.

In addition, an analysis of the frequency of swimming across the platform during the 60 s observation period revealed that there were significant differences among the Sham, MCAO, and rTMS groups. The frequency of swimming across the platform was lower (*P* = 0.001) in the MCAO group (0.9 ± 0.9) than in the Sham group (3.5 ± 2.2), but it was higher (*P* = 0.045) in the rTMS group (2.5 ± 0.8) than in the MCAO group (Figure 2C). The typical swimming paths observed in the spatial probe trial of each group are shown in Figures 2G–I.

### Effects of rTMS on neurogenesis in the ipsilateral SGZ after focal cerebral ischemia

**Figure 3:**
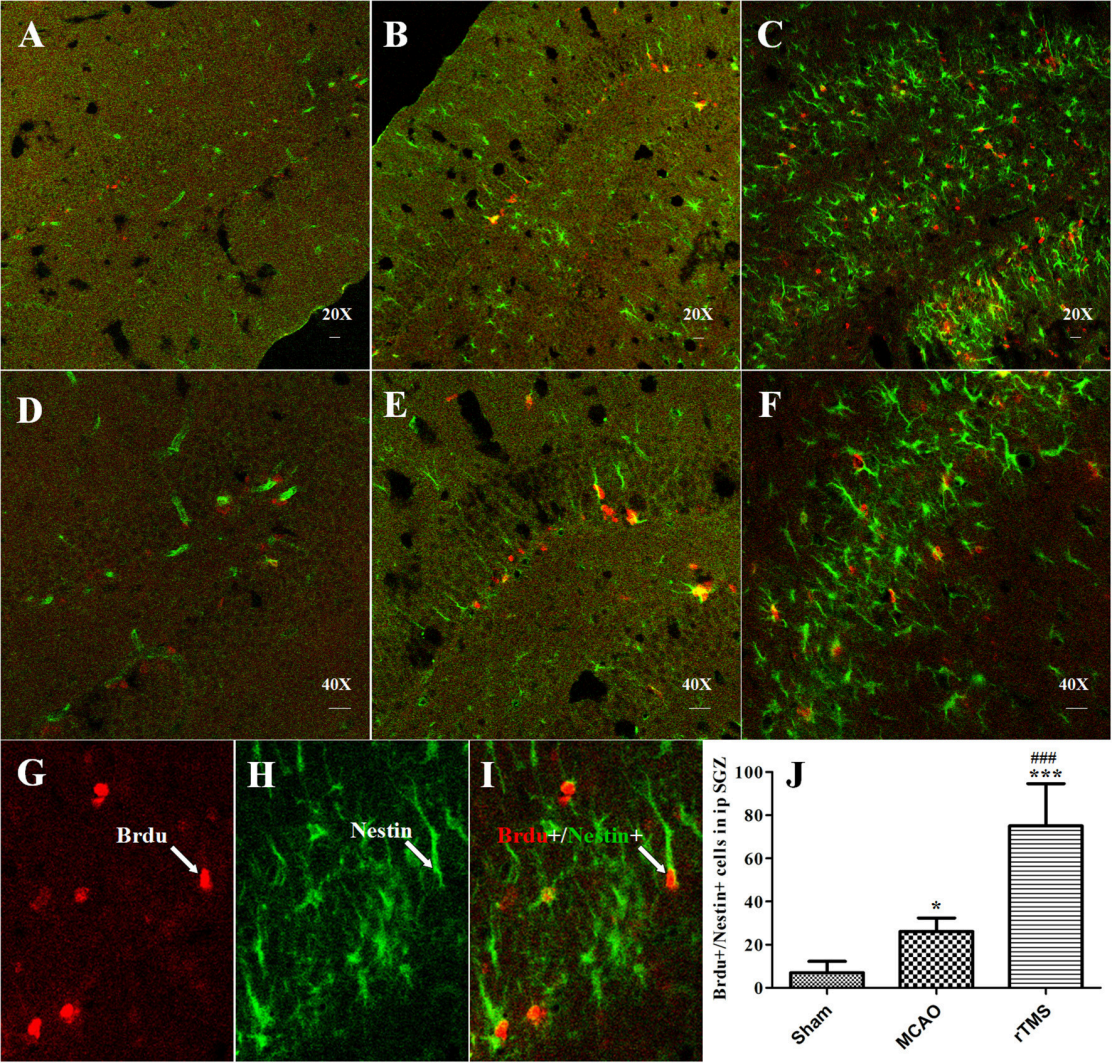
rTMS increased the number of BrdU (red) and Nestin (green) co-immunofluorescence-labeled cells in the ipsilateral SGZ at 7 days after surgery. Panels **(A–F)** show BrdU^+^/Nestin^+^ cells in the ipsilateral SGZ in the Sham **(A,D)**, MCAO **(B,E)**, and rTMS **(C,F)** groups. Confocal images are shown at 20X and 40X magnification (bar = 20 μm). BrdU-positive cells are labeled red **(G)**, Nestin-positive cells are labeled green **(H)**, and BrdU^+^/Nestin^+^ positive cells are double-labeled **(I)**. **(J)** Quantification of the number of BrdU^+^/Nestin^+^ cells in the ipsilateral SGZ at 7 days after surgery. ^*^P = 0.042 and ^***^P < 0.001 vs. the Sham group; ^###^*P* < 0.001 for the rTMS group vs. the MCAO group; SGZ, subgranular zone.

To evaluate the proliferation of NSCs in response to rTMS, double immunofluorescence staining was performed for BrdU and Nestin at 7 days after treatment (Figure 3). The results showed that the number of BrdU^+^/Nestin^+^ cells in the SGZ was higher in the MCAO (26.2 ± 6.24) group and rTMS (75 ± 19.64) group than in the Sham (6.8 ± 5.23) group (*P* = 0.042 and *P* < 0.001, respectively). Moreover, there were more BrdU^+^/Nestin^+^ cells in the rTMS group than in the MCAO group (*P* < 0.001), implying that rTMS facilitated adult NSC proliferation in the SGZ in response to focal cerebral ischemia.

**Figure 4:**
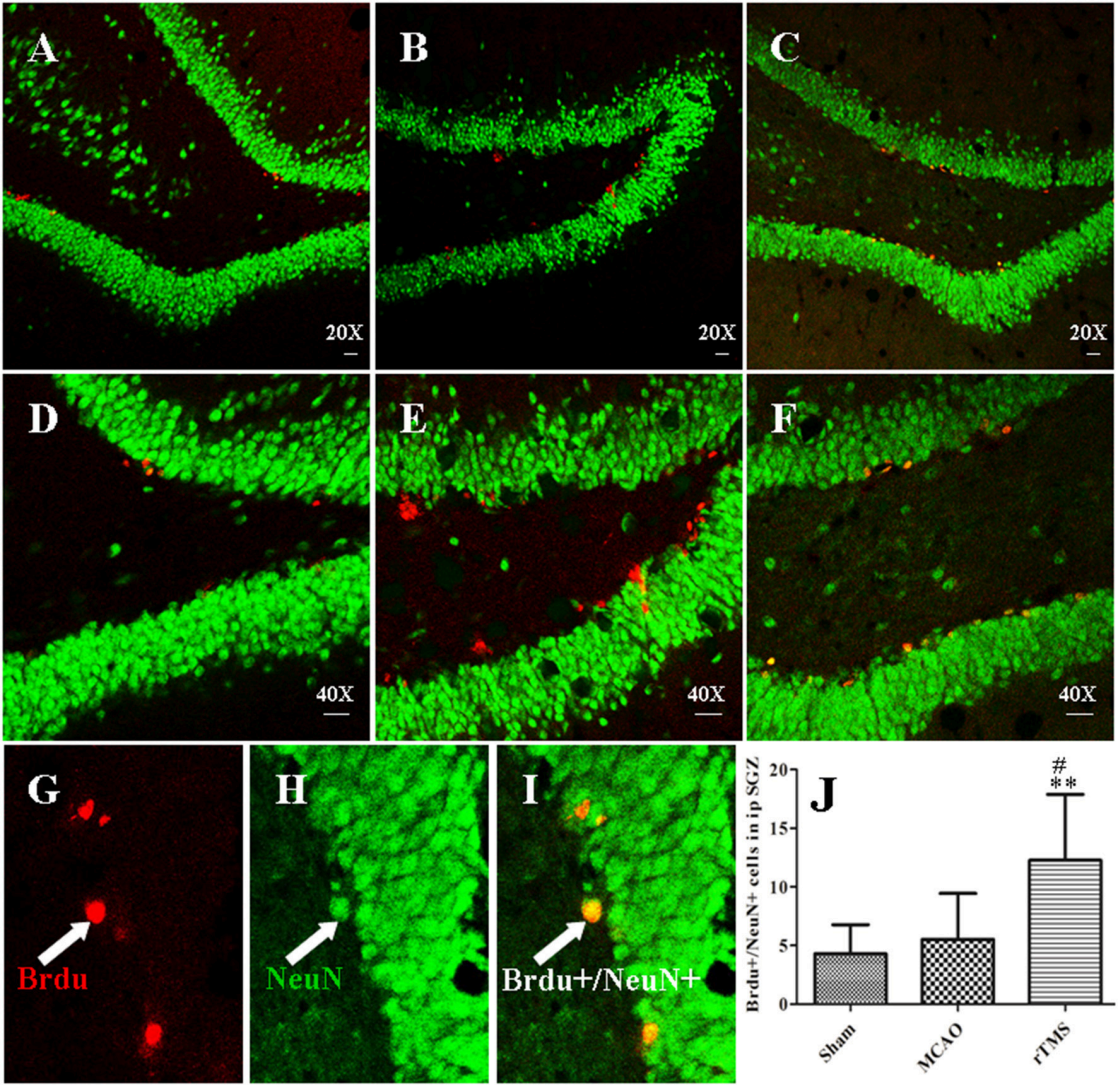
rTMS increased the number of BrdU (red) and NeuN (green) co-immunofluorescence-labeled cells in the ipsilateral SGZ at 14 days after surgery. Panels **(A–F)** show BrdU^+^/NeuN^+^ cells in the ipsilateral SGZ in the Sham **(A,D)**, MCAO **(B,E)**, and rTMS **(C,F)** groups. Confocal images are shown at 20X and 40X magnification (bar = 20 μm). BrdU-positive cells are labeled red **(G)**, NeuN-positive cells are labeled green **(H)**, and BrdU^+^/NeuN^+^ cells are double-labeled **(I). (J)** Quantification of the number of BrdU^+^/NeuN^+^ cells in the ipsilateral SGZ at 14 days after surgery. ^**^P = 0.006 vs. the Sham group; ^#^*P* = 0.021 for the rTMS group vs. the MCAO group; SGZ, subgranular zone.

To further explore the differentiation of newborn cells, which incorporate BrdU, in response to rTMS, we performed double immunofluorescence staining using antibodies against BrdU and a marker of mature neuronal nuclei (NeuN) in the SGZ at 14 days after treatment (Figure 4). We found that there were more BrdU/NeuN double-labeled cells in the SGZ in the rTMS (12.3 ± 5.6) group than in the MCAO (5.5 ± 3.9) group (*P* = 0.021) and the Sham (3.8 ± 2.9) group (*P* = 0.006), indicating that cell proliferation was promoted in the rTMS group. Only a few positive cells were observed in the ipsilateral SGZ at 14 days after MCAO, and there was no significant difference between this group and the Sham group (*P* > 0.05).

### Effects of rTMS on BDNF signaling in the hippocampus after focal cerebral ischemia

**Figure 5:**
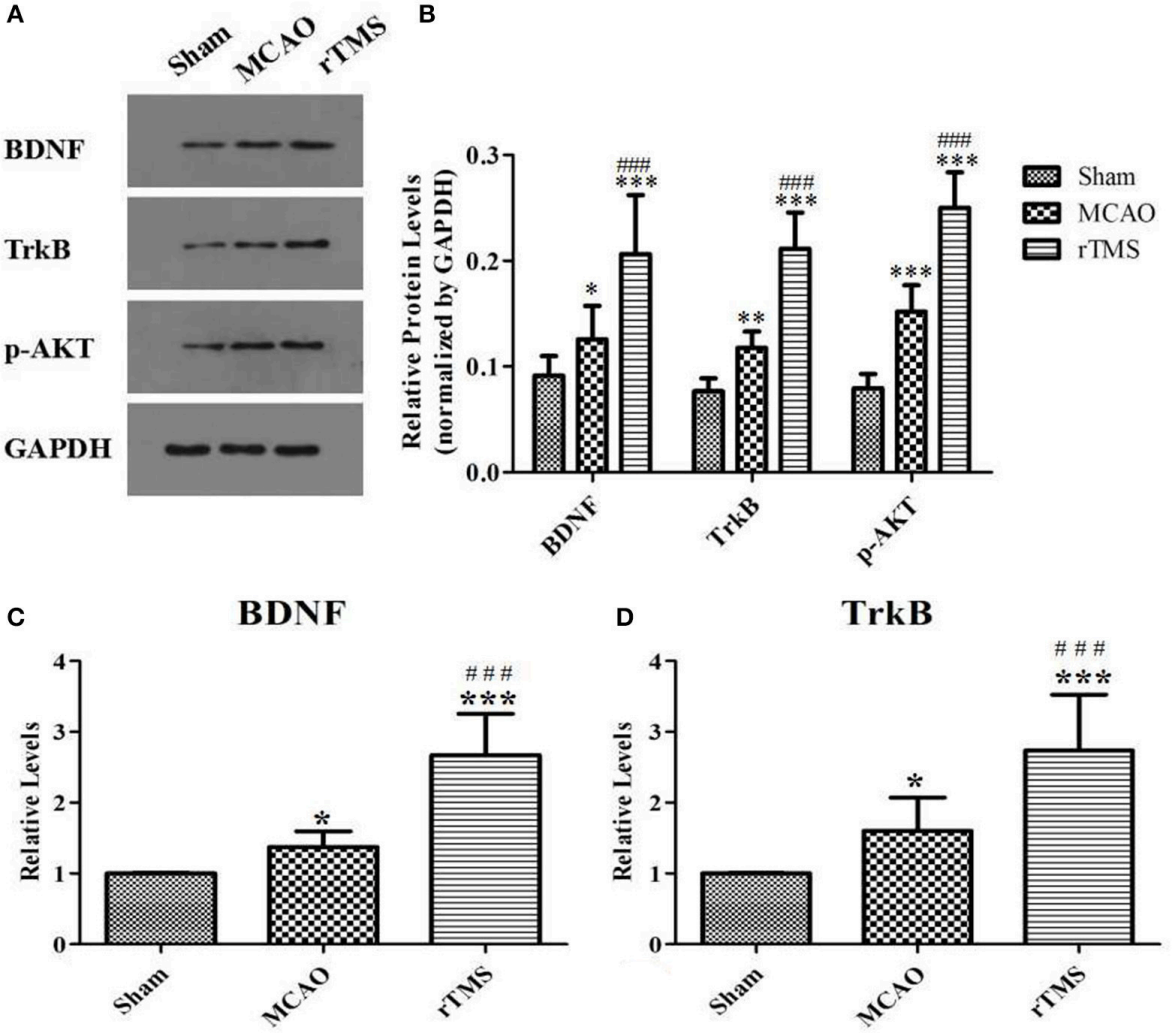
Effects of rTMS on the expression of BDNF pathway members in the hippocampus at 14 days after surgery. **(A)** Gel electrophoresis of BDNF, TrkB and p-AKT. **(B)** The ratio of the expression level of the target genes to that of GAPDH in the three groups. **(C)** RT-PCR analysis of BDNF mRNA levels. **(D)** RT-PCR analysis of TrkB mRNA levels. ^*^P < 0.05, ^**^P < 0.01, and ^***^P < 0.005 compared to the Sham group; ^###^*P* < 0.005 for the rTMS group compared to the MCAO group.

To investigate the effect of rTMS on the BDNF pathway, western blot analysis, and RT-PCR were performed to examine the expression of crucial signaling molecules in the ipsilateral hippocampus. As shown in Figure 5, BDNF, TrkB, and p-AKT protein expressions levels were higher in the MCAO group than in the Sham group (*P* < 0.05). Moreover, significantly higher levels of these proteins were observed in the ischemic cerebral tissues of the rTMS group than in those of the MCAO group (*P* < 0.005). In addition, RT-PCR showed that in the rats treated with rTMS, the expression levels of the mRNAs of BDNF and TrkB were also significantly higher than the levels observed in the other groups (*P* < 0.05).

### Effects of rTMS on neuronal apoptosis in the ipsilateral hippocampus after focal cerebral ischemia

**Figure 6:**
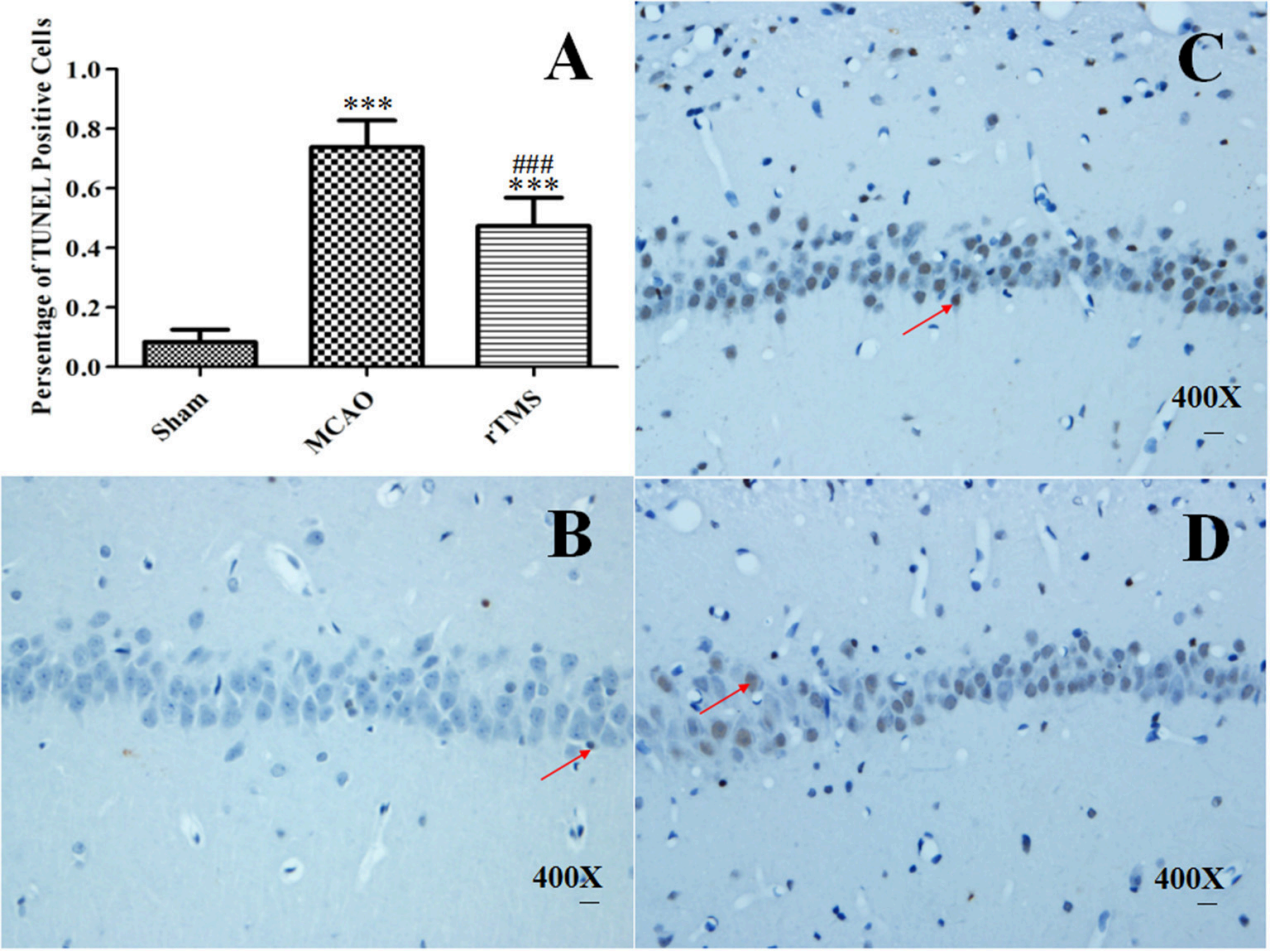
Effect of rTMS on the number of terminal deoxynucleotidyl transferase-mediated dUTP nick end-labeling (TUNEL)-positive cells in the hippocampus. **(A)** Percentage of TUNEL-positive cells in the ipsilateral hippocampus at 14 d post-surgery. The scale bar represents 20 μm. **(B–D)** Representative photomicrographs of the pyramidal cell layer of the CA1 region in the Sham **(B)**, MCAO **(C)**, and rTMS **(D)** groups. ^***^P < 0.005 vs. the Sham group and ^###^*P* < 0.005 for the rTMS group vs. the MCAO group.

**Figure 7:**
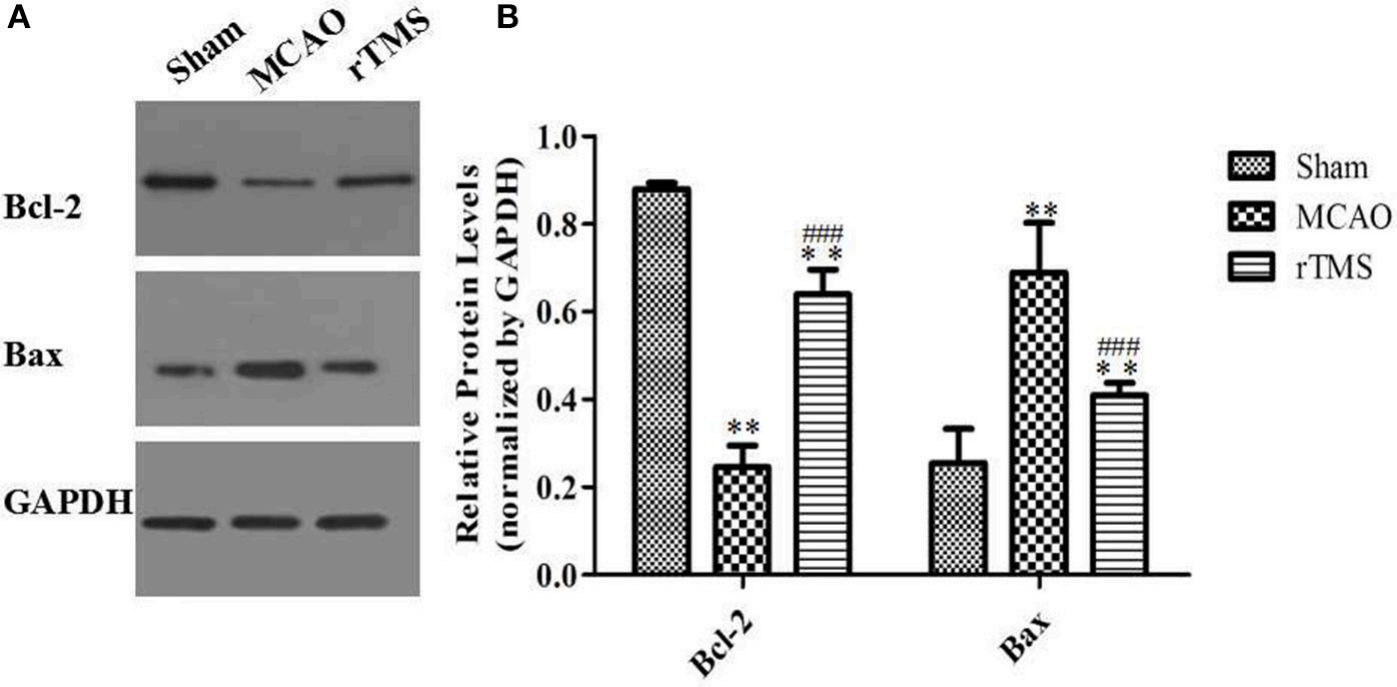
Effects of rTMS on Bcl-2 and Bax expression levels in the hippocampus. **(A)** Gel electrophoresis of Bcl-2, Bax, and GAPDH. **(B)** Relative levels of the Bcl-2 and Bax proteins in the three groups. ^**^P < 0.01 compared to the Sham group. ^###^*P* < 0.005 for the rTMS group compared to the MCAO group.

To determine whether rTMS affects cell survival, we counted apoptotic nuclei and detected the levels of the apoptosis-related proteins Bcl-2 and Bax in the ipsilateral hippocampus after focal cerebral ischemia. Apoptotic neuronal death was evaluated using TUNEL staining (Figure 6), and proteins were detected using western blot analysis (Figure 7).

Our analysis of the whole hippocampus revealed that there were clearly significant signs of neuronal loss in the experimental rats. As shown in Figure 6A, 0.083 ± 0.042% of cells were TUNEL-positive in the Sham group, 0.738 ± 0.091% were positive in the MCAO group, and 0.474 ± 0.095% were positive in the rTMS group (*P* < 0.001 for the MCAO group vs. the Sham group and *P* < 0.001 for the rTMS group vs. the MCAO group). The rate of apoptosis was significantly lower in the rTMS group. Because previous reports have indicated that the CA1 region is more vulnerable to ischemic damage (Walton et al., 1996), we captured representative photomicrographs of the pyramidal cell layer of the CA1 region (Figures 6B–D).

Because the balance between Bcl-2/Bax is involved in the regulation of apoptotic cell death, we next investigated whether rTMS influenced Bcl-2/Bax expression. As shown in Figure 7, Bcl-2 expression was significantly lower and Bax expression was significantly higher in the MCAO rats than in the Sham rats (*P* < 0.01). Moreover, rTMS significantly enhanced the expression of Bcl-2 and significantly reduced the expression of Bax in comparison to the effect observed in the MCAO group (*P* < 0.005). These results demonstrate that neuronal apoptosis is enhanced by the induction of ischemia and that treatment with rTMS significantly inhibited this effect.

## Discussion

Cognitive impairment and memory dysfunction are common symptoms observed in stroke survivors that significantly affect their quality of life. In the present study, we show that 10 Hz rTMS improves cognitive impairment, stimulated neurogenesis and inhibited apoptosis when focal cerebral ischemia was induced in the hippocampus. In addition, we found that the BDNF signaling pathway is a crucial contributor to the effects of rTMS.

rTMS is a promising approach for treating PSCI stroke-induce cognitive impairment. Several previous investigations showed that chronic high-frequency rTMS ameliorated cognitive impairment in normally aging individuals (Guse et al., 2010; Hsu et al., 2015). Mally and Stone demonstrated that rTMS exerted beneficial effects on cognitive functions and memory in patients with central nervous system diseases (Mally and Stone, 2007). In addition, evidence in recent studies has shown that rTMS may significantly improve cognitive impairment in stroke patients. For example, Lu et al. showed that low-frequency rTMS improved cognitive and memory functions in patients with stroke and that this affect lasted for 2 months after treatment (Lu et al., 2015). Park et al. used K-MMSE and LOTCA-G scores to show that cognitive functions were significantly improved by rTMS in stroke patients (Park and Yoon, 2015). Finally, it was suggested in a recent systematic review that rTMS exerts a positive effect on cognitive impairment in stroke patients (Nouhaud et al., 2017). Consistent with previous clinical results, we used a rat model of MCAO to confirm that rTMS improves cognitive impairment.

Although, previous studies have shown that rTMS exerts positive effects on cognition, its clinical effects have remained the subject of dispute (Kim et al., 2010). A variety of technical variables, such as the stimulation frequency applied, its location, the type of coils used, the duration and number of treatments, and the gap between procedures might contribute to the variation observed across the results of different studies. Low- and high-frequency rTMS have yielded inconsistent results, and data from previous studies have not resolved the controversy regarding which is the best frequency to use. However, high-frequency rTMS does seem to achieve better outcomes in some patients and models. For example, a study of post-traumatic stress syndrome showed that better outcomes were achieved by high-frequency than low-frequency rTMS (Boggio et al., 2010). In Alzheimer's disease, better cognitive functions were achieved following the application of high-frequency than low-frequency rTMS (Ahmed et al., 2012). On et al. reported that working memory showed improved accuracy following treatment with 1,000 rTMS sessions at 10 Hz and 100% of the exercise intensity threshold (Ohn et al., 2008). Rektorova et al. reported that 10 Hz rTMS resulted in a mild but significant stimulatory effect in executive functions in cerebrovascular disease patients (Rektorova et al., 2005). In the current study, we applied rTMS at a frequency of 10 Hz and found that some of the treated rats exhibited noticeable behavioral changes, perhaps because of the low intensity used in our treatment. In addition, the results of our study confirm that 10 Hz rTMS is safe and effective for improving PSCI.

Despite the potential of rTMS, the specific mechanisms by which it improves PSCI have not been investigated. Neurogenesis may restore cognitive functions that are impaired by ischemic stroke, and an increasing amount of evidence indicates that a sufficient number of neurons are required for normal hippocampal functions. Hence, we sought to determine the mechanism by which 10 Hz rTMS affects cognitive impairment in MCAO rats. Our results incorporate several lines of evidence demonstrating that treatment with rTMS increases neurogenesis and decreases apoptosis in the hippocampus. First, the results of BrdU^+^/Nestin^+^ immunofluorescence staining show that applying 10 Hz rTMS to MCAO rats induced proliferation in NSCs in the SGZ. Second, the results of double immunostaining for BrdU and NeuN show that applying 10 Hz rTMS to MCAO rats enhanced differentiation in newborn cells in the SGZ, and these new neurons were integrated into the existing hippocampal circuitry. Finally, TUNEL labeling revealed that treatment with 10 Hz rTMS down-regulated neuronal apoptosis. All of the above data support the notion that rTMS is an efficient therapeutic treatment for patients with cognitive impairment and that the effects of rTMS on these symptoms may involve increasing neurogenesis and suppressing apoptosis in the hippocampus. To our knowledge, this is the first study to explore the effects of and mechanisms underlying rTMS in PSCI. Nevertheless, our results are in agreement with the results of previous studies that have indicated that rTMS plays an important role in both neurogenesis and apoptosis. Ueyama et al. reported that chronic rTMS increased hippocampal neurogenesis in healthy rats (Ueyama et al., 2011). In addition, Feng et al. demonstrated that high-frequency rTMS promoted cellular proliferation in the hippocampus in depression models (Feng et al., 2012). In addition, Yoon et al. observed that in cerebral ischemia, rTMS exerted an anti-apoptotic effect in the peri-ischemic area (Yoon et al., 2011). Thus, the neurogenesis-inducing and apoptosis-inhibiting effects of rTMS represent potential means for the recovery of cognitive functions following ischemic injury. However, determine how the normal regulatory pathways underlying adult neurogenesis and apoptosis are altered by rTMS is essential to any attempt at manipulating rTMS as a therapeutic treatment for stroke.

In some studies, it has been proposed that rTMS supports improved memory formation and motor learning by enhancing activity at the level of neurotransmitters or neurotrophic factors. Wang et al. reported that rTMS improved the restoration of cognitive ability and exerted a neuroprotective effect in vascular dementia rats and that this effect may have been the result of increases in the levels of BDNF, TrkB, and SYN in the CA1 region (Wang et al., 2015). We observed that the protein and mRNA levels of vital members of the BDNF signaling pathway are higher in the ipsilateral hippocampus after rTMS. The BDNF signaling pathway is closely associated with cerebral ischemia and neurogenesis and plays important roles in supporting neuronal survival and maintenance (Wei et al., 2015). Evidence presented in several studies has revealed that increasing BDNF levels may be a promising therapeutic approach for restoring motor and cognitive functions, and numerous studies have firmly supported the notion that normal hippocampal BDNF levels are required for proper cognitive function (Goldberg et al., 2015). Thus, manipulating the BDNF pathways represents a viable approach to treating a variety of neurological and psychiatric disorders. Consistent with the majority of published studies (Jeong et al., 2014; Yin et al., 2015), our results indicate that BDNF plays an inhibitory role in the regulation of neurogenesis and neuron survival. Therefore, the up-regulation of BDNF signaling by rTMS could potentially affect the number and survival rate of neurons in the neurogenic niche. We have shown that the rTMS–induced up-regulation of BDNF signaling exerts neuroprotective functions that may improve PSCI.

The major aim of the present study was to investigate the mechanisms underlying the neuroprotective efficacy of rTMS in PSCI. Increasing our understanding of the environment in the neurogenic niche and the mechanisms involved in maintaining it are paramount to efforts aimed at enhancing endogenous recovery processes. We show that 10 Hz rTMS promotes neurogenesis and inhibits neuronal apoptosis in the ipsilateral hippocampus after focal cerebral ischemia. We also demonstrate that the expression levels of BDNF signaling components are increased and that the expression levels of apoptosis-related proteins are altered in the ischemic hippocampus after rTMS. These results suggest that the cognitive recovery induced by rTMS could be enhanced by manipulating extracellular factors in the hippocampus that are associated with these neurotrophic factors. Additional factors known to be important during these processes should also be explored to further clarify the neuroprotective role of rTMS in PSCI.

In conclusion, the results presented in this study indicate that rTMS improves PSCI, promotes neurogenesis and suppresses neuronal apoptosis in the hippocampus of adult rats with focal cerebral ischemia. Moreover, the mechanisms underlying the neuroprotective effects of rTMS might be associated, at least in part, with the activation of the BDNF signaling pathway. These results collectively suggest that up-regulating BDNF signaling using rTMS affects neurogenesis and apoptosis in ischemic hippocampus and is essential for improving PSCI.

## Author contributions

XLH and XHH contributed to the research design and data analysis. FG carried out the experiment, analyzed the data and wrote the manuscript. JL and YD participated in the experiment of the behavior test, rTMS treatment and immunostaining. All authors read and approved the final manuscript.

### Conflict of interest statement

The authors declare that the research was conducted in the absence of any commercial or financial relationships that could be construed as a potential conflict of interest.
